# The effect of motivational interviewing on smoking cessation intention in patients with lung cancer: A randomized controlled trial

**DOI:** 10.18332/tid/219211

**Published:** 2026-08-01

**Authors:** Sibel Dogru, Sema Aytaç, Özlem Ovayolu

**Affiliations:** 1 Faculty of Medicine, Department of Pulmonary Diseases, Gaziantep University, Gaziantep, Türkiye; 2 Nursing Department, Gaziantep University, Gaziantep, Türkiye

**Keywords:** motivational interviewing, smoking cessation intention, lung cancer, real‑time WhatsApp^®^ video calling

## Abstract

**INTRODUCTION:**

This randomized controlled trial investigated the effect of motivational interviewing (MI) on smoking cessation intention in patients diagnosed with lung cancer.

**METHODS:**

Patients with lung cancer enrolled in the study were divided into intervention (n=21) and control (n=21) groups based on random sampling after power analysis. Patients in the intervention group received two MI sessions through real-time WhatsApp^®^ video calling, administered by the researcher on day 1 of the study and 15 days later. The control group received standard care. At baseline, both groups completed a baseline questionnaire, the Smoking Cessation Intention Scale (SCIS), the Fagerström test for nicotine dependence (FTND), and the Eastern Cooperative Oncology Group (ECOG) performance status scale. At the end of the study, all scales except the baseline questionnaire were re-administered to both groups.

**RESULTS:**

Post-intervention, the control group exhibited significantly lower SCIS scores than the intervention group (17.19 ± 4.7 vs 23.23 ± 4.5, p<0.001). The control group also demonstrated a significant decline in SCIS scores from pre- to post-test (22.90 ± 5.3 vs 17.19 ± 4.7, p<0.001). In the intervention group, mean FTND scores decreased substantially following MI (6.2 ± 2.5 vs 2.5 ± 2.0, p=0.012), whereas the control group showed a small, non-significant reduction in FTND scores (5.6 ± 3.1 vs 4.9 ± 3.2, p=0.178). ECOG performance scores remained stable in both groups across the study period.

**CONCLUSIONS:**

The findings indicate that two sessions of MI delivered via real-time WhatsApp^®^ video calling did not improve smoking cessation intention in patients with lung cancer. However, it reduced nicotine dependence levels. These findings support the development of personalized cessation programs to bolster quit intentions in this high-risk population.

**CLINICAL TRIAL REGISTRATION:** The study is registered on the official website of ClinicalTrials.gov

**IDENTIFIER:** NCT06912113

## INTRODUCTION

Tobacco dependence remains one of the foremost causes of preventable disease and death globally^1^. Comprising more than 4000 chemical compounds, cigarette smoke is a potent carcinogen that induces malignancies across multiple organ systems, predominantly affecting the lungs^1^. Evidence suggests that tobacco use is responsible for approximately 40% of all cancer diagnoses in the United States^2^.

Intention to quit smoking is a key determinant of cessation behavior. However, quit intentions vary substantially across populations. While approximately 70% report some degree of receptiveness toward quitting, 13% of those undergoing lung cancer screening indicate no intention to quit at all^3,4^. This variability underscores the importance of systematically assessing quit intention to identify individuals at risk for continued smoking and to tailor cessation interventions^3,4,5-8^. Moreover, patients with lung cancer face considerable barriers to participating in in-person cessation programs – including symptom burden, treatment-related toxicity, reduced mobility, and geographical distance – highlighting the need for remotely delivered approaches^9^.

A range of pharmacological and non-pharmacological smoking cessation strategies is available^1^. Among behavioral approaches, motivational interviewing (MI) is a client-centered, evidence-based counseling method designed to strengthen intrinsic motivation for behavior change^10-14^. However, recent evidence suggests that research on the intention to quit smoking remains insufficiently examined, with oncology research largely emphasizing cessation and survival outcomes^15^. Considering this gap, quit intention represents a clinically meaningful and feasible intermediate outcome. In patients with advanced lung cancer – who often experience limited survival and significant challenges in achieving cessation – enhancing quit intention may represent a practical and impactful target for promoting future quit attempts.

Motivational Interviewing (MI) can be delivered through various digital modalities, including chatbots and video-based communication^4,16,17^. Video-based delivery – such as WhatsApp^®^ video calling – offers an accessible, secure, and efficient way to support clinical communication, particularly for patients who may have difficulty attending in-person visits. Real-time visual interaction allows clinicians to attend to patients’ facial expressions and other nonverbal cues, promoting clearer understanding and a more supportive therapeutic connection^18^. Although video-based MI has demonstrated feasibility and acceptability in other populations, to our knowledge, no studies to date have examined whether

WhatsApp^®^-delivered MI can enhance smoking-cessation intention among patients with lung cancer^19^.

Given the limited evidence on quit intention in this context, evaluating remotely delivered MI may offer meaningful insights into how best to support tobacco cessation efforts in this population.

Therefore, the primary objective of this randomized controlled trial was to determine whether motivational interviewing delivered via WhatsApp^®^ video calling increases smoking cessation intention among patients with lung cancer compared with standard care.

## METHODS

This randomized controlled trial was conducted between 29 March and 31 December 2024, at the Department of Pulmonary Diseases, Research and Application Hospital, in a single-center setting, and reported in accordance with the CONSORT guidelines (The CONSORT checklist is available in the Supplementary file). Eligible patients with a confirmed diagnosis of lung cancer were allocated to the intervention or control group using simple random sampling. *A priori* power analysis performed with G*Power 3.1 (α=0.05, power=0.80) indicated a minimum sample size of 21 participants per group. Accordingly, 42 patients (21 intervention group and 21 control group) were enrolled in the trial (Supplementary file Figure 1). No interim analyses or stopping guidelines were planned or conducted.

**Figure 1:**
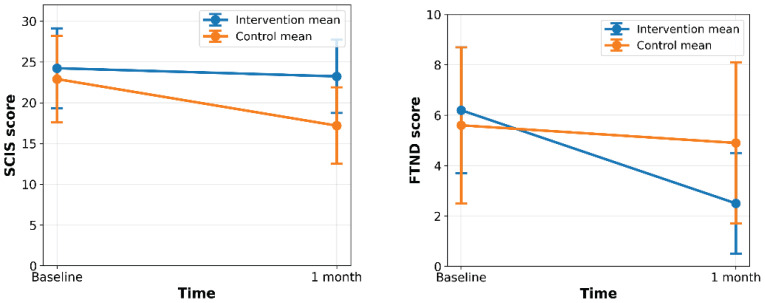
Changes in SCIS and FTND scores in intervention and control groups, randomized controlled trial, March–December 2024, Research and Application Hospital, Türkiye (N=42)

### Participants

The study sample included individuals who met the eligibility criteria and provided informed consent. Inclusion criteria were: age ≥18 years; histologically confirmed lung cancer; lifetime consumption of ≥100 cigarettes; at least one cigarette smoked in the past month; ability to communicate without assistance; and internet access. And classification within the first three stages of the Transtheoretical Model of Behavior Change (TTM)^20^ (1. Precontemplation: No intention to quit or recognition of smoking as a problem, 2. Contemplation: Considering quitting and gathering information on health risks and cessation methods, and 3. Preparation: Intending to quit within the next month). This classification, in the present study, was utilized solely as a baseline descriptor and an inclusion criterion, rather than as a longitudinal outcome measure^20^.

Exclusion criteria were: occasional smokers; current participation in other cessation programs; use of any cessation pharmacotherapy or other addictive substances; and use of non-cigarette tobacco products. Eleven patients were excluded from the study based on these criteria. Furthermore, 28 patients were excluded for failing to meet the inclusion criteria.

### Instruments and tests

Data were collected using the baseline questionnaire, the Smoking Cessation Intention Scale (SCIS), the Fagerström test for nicotine dependence (FTND), and the Eastern Cooperative Oncology Group (ECOG) scale.

### Baseline questionnaire form

This form consists of two sections that gather sociodemographic data and disease and smoking-related information. The first section includes sociodemographic characteristics (age, sex, and education level), pathological diagnosis, presence of metastasis, and comorbidities. The second section includes smoking initiation age, total smoking duration, daily cigarette count, pack-years (calculated as [daily cigarettes/20] × years smoked), number of prior quit attempts, use of pharmacological cessation treatments, fear of quitting, number of quit attempts in the past 12 months, and post-intervention change in cigarette consumption (increased, unchanged, or decreased compared to baseline).

### Smoking Cessation Intention Scale (SCIS)

This scale was developed by Söyler et al.^21^ to measure smoking cessation intention in smokers and consists of eight items. Scoring is performed by summing the scores for each item. The scale includes the following questions: ‘I plan to quit smoking’, ‘I will try to quit smoking’, ‘I fantasize about quitting smoking’, ‘I want to quit smoking’, ‘Quitting smoking is important to me’, ‘I will quit smoking soon’, ‘I am researching methods for quitting smoking’, and ‘I intend to consult a healthcare professional about quitting’. The scale uses a 5-point Likert response [1 (strongly disagree) to 5 (strongly agree)]. The Cronbach’s alpha value of the original scale was 0.929. Total scores range from 8 to 40, with higher scores indicating stronger intention^21^. In the present study, the Cronbach’s alpha value of the scale was calculated as 0.872.

### Fagerström test for nicotine dependence (FTND)

This scale, developed by Heatherton et al.^22^ to assess physical nicotine dependence, was validated in Turkish by Uysal et al.^23^. The scale consists of six items, each scored between 0 and 3. Total scores range from 0 to 10. In this study, scores of 0–4 were classified as ‘low dependence’, 5–6 as ‘moderate dependence’, and ≥7 as ‘high dependence’. The Cronbach’s alpha value of the scale was 0.681.

### Eastern Cooperative Oncology Group (ECOG) scale

ECOG scale developed by Zubrod et al.^24^ to assess physical performance in cancer patients, this scale assigns scores from 0 to 5 based on daily activity levels: 0 (asymptomatic), 1 (symptomatic but fully ambulatory), 2 (symptomatic but capable of self-care), 3 (bedridden >50% of the day), 4 (completely bedridden), and 5 (deceased).

Baseline questionnaire form, SCIS, FTND, and ECOG scale, were administered at the beginning of the study. At the end of the study, all scales except the baseline questionnaire were re-administered to both groups.

### Blinding

Owing to the nature of the intervention, the practitioner could not be blinded. The participants remained blinded to the group allocations.

### Intervention procedure

At the beginning of the study, participants in the intervention arm completed the baseline questionnaire and the SCIS, FTND, and ECOG scales. At the end of the study, the SCIS, FTND, and ECOG scales were re-administered, and current smoking status was recorded using the baseline questionnaire.

They then received two MI sessions via real-time WhatsApp^®^ video calls – immediately after baseline assessment and 15 days later – each lasting approximately 25–30 minutes^25^.

This remote video consultation method was selected to enable real-time visual interaction with this vulnerable patient population, thereby enhancing the accessibility and continuity of counseling while maintaining fidelity to the core principles of MI.

In this study, the stages of the Transtheoretical Model of Behavioral Change were used only as a conceptual reference to determine the content and order of the interview questions. MI constituted the primary methodological approach and was implemented in accordance with its core principles. Importantly, MI was employed not as a mechanism to guide participants through predefined stages of change, but as a flexible, participant-centered framework to direct the focus of interviews^10,20^. Motivational interviewing was delivered consistently with its foundational spirit of partnership, acceptance, compassion, and evocation, while incorporating core MI principles of expressing empathy, developing discrepancy, and supporting self-efficacy sessions focused on empathic understanding, collaborative exploration of ambivalence, and the elicitation and reinforcement of change talk. Discrepancies between current smoking behavior and personal goals were explored to enhance intrinsic motivation, while resistance was addressed through reflective listening and non-confrontational strategies. Self-efficacy was actively supported throughout. To initiate the conversation about change, the following topics were discussed: the pros and cons of change, the intention and ability to change, the reasons for change, and plans and goals related to change. To elicit change talk, several strategies were used: exploration of the pros and cons of change and elaboration of reasons for change by asking ‘What else?’ to assess optimism about change, questions such as ‘If you’ve decided to make a change, what makes you think you can do it?’ were asked. Additionally, hypothetical questions about change, such as ‘If you decided to change, what do you think would help?’, and change-intention questions, such as ‘What needs to change?’ and ‘What do you think you could do?’, were asked. Regarding the plans, they were asked, ‘What are you planning to do, what are your options?’. MI-consistent techniques – including open-ended questions, affirmations, reflective listening, summarizing, and asking permission before providing information – were used^10,^^26,27^. A detailed description of the process is provided in Supplementary file Table S1^10,26,27,28^.

### Control group

At the beginning of the study, participants in the control group completed the baseline questionnaire and the SCIS, FTND, and ECOG scales. Patients in the control group were informed during outpatient visits, as per hospital policy, that they needed to quit smoking. No intervention was performed on this group. Afterward, 1 month later, they were reassessed using their scales via WhatsApp^®^ video calling.

### Outcome measures

The primary outcome was smoking cessation intention at 1 month post-intervention, as measured by the SCIS. Secondary outcomes were changes in nicotine dependence (FTND), functional performance (ECOG), and daily cigarette consumption.

### Ethical considerations

Ethical approval was obtained from the Clinical Research Ethics Committee (Approval No: 2024/92, Date: 27.03.2024). All participants were informed about the study, and verbal consent was obtained before participation. The study was conducted in accordance with the Declaration of Helsinki.

### Statistical analysis

Data were analyzed using IBM SPSS Statistics 22 (IBM Corp., Armonk, NY, USA). Normality was assessed via the Shapiro–Wilk test. Descriptive data were presented as frequencies, percentages, means, and standard deviations. Within-group pre-post changes were analyzed using Wilcoxon signed-rank tests, while between-group comparisons of change scores and numerical data were assessed using Mann–Whitney U tests. Categorical variables were compared using the chi-squared test. All randomized participants completed the study and were included in the analyses within their original groups. All participants were analyzed according to the intention-to-treat principle. All statistical tests were two-tailed, and a p<0.05 was considered statistically significant.

## RESULTS

### Comparison of patient characteristics

The mean age of the patients was 62.47 ± 11.78 and 62.76 ± 12.86 years for the intervention and control groups, respectively. All patients in the intervention group were men, and most were primary school graduates. In the control group, 85.7% of the patients were men, and 6.2% had primary education (Table 1). No statistically significant baseline differences between groups were observed for any parameter, except pathological diagnosis (p>0.05).

**Table 1:** Comparison of baseline characteristics between intervention and control groups among patients with lung cancer, randomized controlled trial, March–December 2024, Research and Application Hospital, Türkiye (N=42)

*Characteristics*	*Intervention* *group* *(N=21)* *n (%)*	*Control group* *(N=21)* *n (%)*	*p*
**Age** (years), mean ± SD	62.47 ± 11.78	62.76 ± 12.86	0.941
**Sex**			
Female	0 (0)	3 (14.3)	0.072
Male	21 (100)	18 (87.5)	
**Education level**			0.668
No formal education	1 (4.8)	2 (9.5)	
Primary education	17 (81)	16 (76.2)	
High school	3 (14.3)	2 (9.5)	
University or higher	0 (0)	1 (4.8)	
**Pathological diagnosis**			0.041
Non-small cell carcinoma	20 (95.2)	14 (66.7)	
Small cell carcinoma	0 (0)	5 (23.8)	
Extrapulmonary carcinoma metastasis	1 (4.8)	2 (9.5)	
**Metastasis**			0.292
Absent	4 (19)	7 (33.1)	
Present	17 (81)	14 (66.7)	
**COPD**			0.753
Absent	13 (61.9)	12 (57.1)	
Present	8 (38.1)	9 (42.9)	
**Hypertension**			0.355
Absent	10(47.6)	12 (57.1)	
Present	12 (57.1)	9 (42.9)	
**Diabetes mellitus**			0.533
Absent	10 (47.6)	8 (38.1)	
Present	11 (52.4)	13 (61.9)	
**Congestive heart failure**			0.214
Absent	16 (76.2)	19 (90.5)	
Present	5 (23.8)	2 (9.5)	
**Age at smoking initiation** (years), mean ± SD	16.95 ± 4.79	18.42 ± 4.22	0.296
**Total duration of smoking** (years), mean ± SD	45.42 ± 12.88	44.28 ± 15.20	0.980
**Cigarettes smoked per day,** mean ± SD	20.80 ± 9.09	26.33 ± 14.59	0.138
**Cigarette smoking** (pack-years), mean ± SD	45.45 ± 19.9	60.95 ± 43.20	0.427
**Smoking cessation attempt**			0.317
No	5 (23.8)	8 (38.1)	
Yes	16 (76.2)	13 (61.9)	
**Fear of quitting smoking**			0.378
No	2 (9.5)	4 (19)	
Yes	19 (90.5)	17 (81)	
**Quitting attempt in the last 12 months**			0.533
No	11 (52.4)	13 (61.9)	
Yes	10 (47.6)	8 (38.1)	

### Smoking‑ and performance‑related outcomes

Post-intervention, the control group exhibited significantly lower SCIS scores than the intervention group (17.19 ± 4.7 vs 23.23 ± 4.5, p<0.001). The control group also demonstrated a significant decline in SCIS scores from pre- to post-test (22.90 ± 5.3 vs 17.19 ± 4.7, p<0.001). In the post-test, FTND scores increased in the control group but decreased in the intervention group compared with baseline (p=0.012 and p<0.001, respectively) (Figure 1). No significant change in ECOG score was found between the post-test and the baseline (Table 2).

**Table 2:** Comparison of smoking-related outcomes and performance status between intervention and control groups before and after the intervention, randomized controlled trial, March–December 2024, Research and Application Hospital, Türkiye (N=42)

*Parameters*	*Intervention* *(N=21)* *Mean ± SD*	*Control* *(N=21)* *Mean ± SD*	*p ^a^*
**SCIS score**			
Baseline	24.23 ± 4.9	22.90 ± 5.3	0.464
1 month later	23.23 ± 4.5	17.19 ± 4.7	**<0.001**
p-value (intra-group)^b^	0.165	**<0.001**	
**FTND score**			
Baseline	6.2 ± 2.5	5.6 ± 3.1	0.560
1 month later	2.5 ± 2.0	4.9 ± 3.2	**0.012**
p-value (intra-group)^b^	**<0.001**	0.178	
**ECOG performance score**			
Baseline	1.57 ± 1.07	1.71 ± 1.14	0.742
1 month later	1.47 ± 1.16	1.71 ± 1.05	0.566
p-value (intra-group)^a^	0.414	1.000	
**Smoking status at the end of the intervention**, n (%)			
No change in smoking at the final evaluation compared with baseline*	8 (38.1)	18 (85.7)	**0.005**
Reduced smoking at the final evaluation compared with baseline*	8 (38.1)	1 (4.8)	
Quit smoking	5 (23.8)	2 (9.5)	

SCIS: Smoking Cessation Intention Scale. FTND: Fagerström test for nicotine dependence. ECOG: Eastern Cooperative Oncology Group performance status scale.

a Mann–Whitney U test.

b Wilcoxon test.

* p<0.005

At the end of the study, the proportions of participants whose daily cigarette consumption remained unchanged, decreased, or reached zero were 8 (38.1%), 8 (38.1%), and 5 (23.8%) in the intervention group, and 18 (85.7%), 1 (4.8%), and 2 (9.5%) in the control group, respectively.

No adverse effects related to the intervention were reported during the study period.

## DISCUSSION

In the present study, a significant reduction in mean SCIS scores was observed in the control group between baseline and follow-up at 1 month. Although the intervention group did not achieve the expected improvement in SCIS scores post-MI, a marked decline in SCIS scores was noted in the control group. This finding suggests that two MI sessions delivered through the two real-time WhatsApp^®^ video calls did not increase the intention to quit smoking in lung cancer patients, but reduced the decrease in intention in the intervention group.

There are several studies that examine the effect of MI on smoking dependence^17,28^. However, research specifically examining MI’s impact on cessation intention remains limited. The present study addresses this gap by evaluating MI’s role in modifying cessation intention. Collby et al.^29^ reported that MI significantly improved cessation intention among adolescents compared with controls. A recent study comparing chatbot-delivered MI and confrontational counseling found similar cessation rates between groups and highlighted MI’s statistically significant effect on cessation intention^30^. Studies often stratify outcomes based on the presence or absence of baseline intention to quit smoking^13,31^. For instance, in one study, MI showed no short-term effect on cessation rates among smokers with the intention of quitting but exhibited negative long-term impacts^13^. Another study reported that MI was more effective for adolescents with low baseline intention of quitting than those with high baseline intention^31^. In the present study, the intervention group demonstrated higher cessation intention than the control group post-MI, underscoring MI’s potential as a multifaceted strategy to strengthen intention. These findings support the integration of MI into smoking cessation treatments. By strengthening intentions to quit and improving adherence to cessation treatments, MI could enhance overall success rates. Future clinical trials should explore longer MI interventions and mixed-methods approaches to unravel underlying motivational mechanisms.

Regarding nicotine dependence, the intervention group showed a significant reduction in FTND scores at 1 month compared with the control group. Similarly, Cevik et al.^32^ evaluated the effect of MI on pregnant women and observed no significant difference in FTND scores at 4 months. However, a significant decrease in FTND scores was observed in the intervention group compared with the control group at 6 months post-MI.

Çavuşoğlu et al.^33^ also reported reduced FTND scores at 6 months among patients with chronic obstructive pulmonary disease (COPD) following MI. In the present study, the rapid and significant decrease in FTND scores at 1 month post-MI intervention compared with the control group is a noteworthy observation. This suggests that MI reduces nicotine dependence among patients with lung cancer, even within 1 month.

A study found that MI shortens the duration of smoking cessation and facilitates smoking cessation^34^. Lindqvist et al.^12^ suggested that MI may be more effective than standard telephone counseling for smoking cessation. However, in the present study, smoking cessation rates were similar between the intervention group and the control group post-MI interventions. These differences may stem from variations in patient populations, intervention intensity, and delivery methods.

Although MI can enhance health awareness, treatment adherence, and overall well-being among patients^10^, it may indirectly have a significant impact on the ECOG performance score. However, in the present study, no significant change in ECOG performance scores was observed 1 month after MI. This suggests that performance status gains in patients with lung cancer may require more prolonged intervention or follow-up.

### Limitations

This study has several limitations. First, although the sample size was modest, it was adequate to detect clinically meaningful effects, as demonstrated by the large effect size (Cohen’s d=1.31; 95% CI: 0.64–1.98) and high statistical power (>99%) in *post hoc* power analysis. However, our findings need to be confirmed in larger cohorts of patients. Second, the single-center design restricts external validity, and the findings may not be generalizable to other countries, healthcare settings, or populations with different cultural or educational backgrounds. Third, the short follow-up duration precludes evaluation of long-term smoking cessation outcomes. Fourth, although the MI sessions were delivered by the same trained researcher following a protocol grounded in the framework of Miller and Rollnick10 and other studies, the absence of formal MI fidelity monitoring remains a methodological limitation. Fifth, a minor imbalance in sex distribution was observed between groups, which can occur by chance in small randomized trials and should be considered when interpreting the findings. Sixth, no formal adjustment for type 1 error was applied; this should be considered in the result interpretation. Future studies with larger samples could incorporate such adjustments. Seventh, a 1-month follow-up was designed to assess MI’s proximal effect on quit intention. Future studies with extended follow-up durations are needed to examine the persistence of these effects and their association with long-term cessation outcomes. A key strength of this trial is its novel use of a multidimensional intention scale to evaluate MI’s impact on smoking cessation intent in patients with lung cancer.

## CONCLUSIONS

The findings obtained in the present study demonstrate that two MI sessions delivered through real-time WhatsApp^®^ video calling did not increase smoking cessation intention; however, they significantly reduced nicotine dependence in patients with lung cancer. These findings support the development of personalized cessation programs to bolster quit intentions in this high-risk population. Further research employing longer MI interventions and qualitative assessments of intention and motivation is warranted to optimize smoking cessation support in this high-risk population.

## Supplementary Material



## Data Availability

The data supporting this research are available from the authors on reasonable request.
